# Calf health from birth to weaning. II. Management of diarrhoea in pre-weaned calves

**DOI:** 10.1186/2046-0481-64-9

**Published:** 2011-09-14

**Authors:** Ingrid Lorenz, John Fagan, Simon J More

**Affiliations:** 1 Herd Health and Animal Husbandry, UCD School Veterinary Medicine, University College Dublin, Belfield, Dublin 4, Ireland; 2 Department of Agriculture, Fisheries and Food, Regional Veterinary Laboratory, Coosan, Athlone, Co. Westmeath, Ireland; 3 Centre for Veterinary Epidemiology and Risk Analysis, UCD School of Veterinary Medicine, University College Dublin, Belfield, Dublin 4, Ireland

**Keywords:** Calf health, Disease management, Neonatal diarrhoea, Oral rehydration, Continued feeding, Prevention, Eimeriosis

## Abstract

Calfhood diseases have a major impact on the economic viability of cattle operations. The second of this three part review series considers the management of diarrhoeic diseases in pre-weaned calves. In neonatal calf diarrhoea, oral rehydration therapy is the single most important therapeutic measure to be carried out by the farmer and is usually successful if instigated immediately after diarrhoea has developed. Continued feeding of milk or milk replacer to diarrhoeic calves is important, to prevent malnourishment and weight loss in affected calves. Indiscriminative antibiotic treatment of uncomplicated diarrhoea is discouraged, whereas systemically ill calves can benefit from systemic antibiotic treatment for the prevention of septicaemia or concurrent diseases. Ancillary treatments and specific preventive measures are discussed. Eimeriosis has a high economic impact on the farming industries due to direct cost of treatment and calf losses, but especially due to decreased performance of clinically as well as sub-clinically affected animals. Emphasis lies on prophylactic or metaphylactic treatment, since the degree of damage to the intestinal mucosa once diarrhoea has developed, makes therapeutic intervention unrewarding.

## Introduction

Calfhood diseases have a major impact on the economic viability of cattle operations, due to the direct costs of calf losses and treatment and the long term effects on performance [1]. Further, calf health was prioritised as one of the most important animal health issues facing the Irish livestock industry in a recent expert Policy Delphi study conducted on behalf of Animal Health Ireland (AHI) [2]. As part of ongoing AHI work, a group of experts was commissioned to provide evidence-based advice on calf health and disease management to Irish farmers, agricultural advisers and veterinary practitioners. As an initial step, a three-part review series on calf health from birth to weaning has been generated, specifically to provide a scientific evidence base for the development of advisory tools on calf health, and to identify gaps in current knowledge to be filled with targeted research. Even though the envisaged output will be specific for Irish husbandry systems, the scope of the reviews should make them useful for the same purpose elsewhere. The reviews cover both suckler and dairy calf management. However, due to the differences in the nature of these systems, some topics will deal mainly or exclusively with either dairy or suckler calves.

Neonatal calf diarrhoea is recognised worldwide as one of the biggest challenges for both the beef and dairy industries. About one third of US beef cow-calf owners agree that it has an economic impact on their operations [3] and it has constantly accounted for more than 50% of unweaned dairy heifer deaths since 1991 [4]. In Ireland, diarrhoea is the most common cause of death in calves from birth to one month of age submitted for post mortem examination (Regional Veterinary Laboratories - Surveillance Report 2009). Disease prevention, though preferable, is not always possible in intensive calf rearing systems. Appropriate calf management, once diarrhoea has developed, is crucial to avoid further economic losses, animal welfare impact and farmer distress.

The second part of this three part review series concentrates on the management of diarrhoea in pre-weaned calves. The first and third parts focus on general aspects of disease prevention in pre-weaned calves [5] and disease prevention and management with particular reference to calf pneumonia [6], respectively.

## Neonatal calf diarrhoea

Enterotoxic *Escherichia coli, Cryptosporidium parvum*, rotavirus and coronavirus are usually seen as the most common infectious causes of neonatal calf diarrhoea [7]. These infectious agents can also be found in faecal samples from healthy calves and in calves from farms without diarrhoea problem [8-11]. Clinical disease develops due to an unfavourable relation between the resistance of the calf and the infectious pressure. The main management factors with impact on the resistance of the calf are calving management to prevent dystocia, timely provision of adequate amounts of colostrum and appropriate diet thereafter, as previously discussed [5]. The infectious pressure can be lowered through general hygiene in the areas of calving, feeding, housing and in general calf handling.

Enterotoxic *E. coli *usually only cause secretory diarrhoea in the first four days of life. The other common infectious agents involved in neonatal calf diarrhoea cause damage to the intestinal mucosa resulting in mixed malabsorptive and secretory diarrhoea. Even if therapy against the causal pathogens was available this pathophysiological mechanism would make it unlikely that the duration of diarrhoea could be significantly influenced [7]. For this reason replacement of fluid and electrolyte losses remains the single most important treatment measure in uncomplicated calf diarrhoea.

### Oral rehydration therapy

Oral rehydration therapy, originally developed in human medicine for the treatment of cholera, is generally recognised as one of the most significant medical advances of the 20th century [12]. The general requirements for an efficient oral rehydration solution (ORS) are that it should be efficiently absorbed, normalise the extracellular fluid volume and correct acidosis [13].

There are several factors to consider while choosing an appropriate ORS. Since sodium is the osmotic skeleton of the extracellular fluid, it must be present in adequate concentration in ORS. A study comparing three ORS with different sodium concentrations in calves found that the solution with a sodium content of 120 mmol/L corrected dehydration, whereas solutions with much lower concentrations did not [14]. Even though there is little evidence that solutions with sodium concentrations > 130 mmol/L are harmful, it is generally suggested that the sodium concentration for ORS should be between 90 and 130 mmol/L [15]. The critical scientific step facilitating the development of oral rehydration therapy was the discovery of the coupled transport of sodium and glucose [16]. Besides glucose, neutral amino acids (e.g. glycine or glutamine) and volatile fatty acids (e.g. acetate or propionate) have been shown to enhance sodium absorption in the intestines [17,18]. Glucose-to-sodium ratios of 1:1 to 3:1 have been recommended [16,19].

Metabolic acidosis is known as a frequent and potentially severe complication of neonatal calf diarrhoea. Diarrhoea leads to loss of bicarbonate via the faeces, decrease of glomerular filtration of hydrogen ions and accumulation of L-lactate in case of severe dehydration. In addition, the production and absorption of D-lactic acid plays a major role in diarrhoeic calves [20]. This pathophysiological abnormality appears to be more common in ruminants than in other domestic species or in infants [20-24], which suggests that newborn ruminants are more prone to developing severe metabolic acidosis than infants during diarrhoea. This is most probably the reason why the current recommendation on the alkalinising capacity for ORS used in calves (60-80 mmol/L [15]) is considerably higher than that of the current WHO-ORS formula (30 mmol/L [19]. Alkalinising agents commonly used in commercial ORS are bicarbonate and bicarbonate precursors, mainly acetate and propionate. Bicarbonate alkalinises the abomasum to a higher degree than propionate and acetate, thus lowering the non-specific resistance of the calves against bacterial infection [25,26]. The impact of bicarbonate in ORS on milk clotting and calf performance is controversial [26-30]. However, in the absence of contrary evidence, it seems reasonable to avoid the use of bicarbonate-containing ORS less than 2 to 4 hours following milk feeding.

As in humans, the purpose of ORS in diarrhoeic calves is to replace electrolytes and fluids that are lost via the intestines. Therefore, ORS should be given to calves as an extra feed (that is, in addition to each normal milk meal) as soon as diarrhoea is observed [15]. The efficiency of this measure relies on early detection, through thorough observation, of diarrhoeic calves. Oesophageal intubation of ORS produces similar, albeit slightly delayed, resuscitative effects compared to ORS that is suckled. Therefore, oesophageal intubation is recommended for calves that are either anorexic or otherwise not likely to drink from an artificial teat [31]. Milk should not be force-fed to calves that are depressed and not interested in drinking. Force-feeding always leads to dysfunction of the oesophageal groove, so that milk fermented in the reticulorumen can further contribute to metabolic acidosis [20]. Even though there is little experimental evidence about limits for oral rehydration therapy, it is generally accepted that intravenous fluid therapy is indicated in severely depressed, recumbent, severely dehydrated (> 8%) and prolonged anorexic (> 24 h) calves. The principles and techniques of intravenous fluid therapy in calves have recently been reviewed [32].

### Continued milk feeding of the diarrhoeic calf

It has traditionally been recommended that milk feeding is withdrawn from diarrhoeic calves, either for a defined period of time or for as long as diarrhoea persists [33]. However, no scientific evidence is available to suggest that starvation of diarrhoeic calves leads to improved clinical outcomes. Indeed, it is now recognised that milk feeding does not worsen or prolong the course of diarrhoea, despite a somewhat lowered digestive capacity. Rather, withdrawal of milk rapidly results in malnourishment and weight loss [27,34]. Continued milk feeding not only provides the energy required for weight gain and growth throughout the period of diarrhoea, but also provides the nutrients that are necessary for the recovery of the intestinal mucosa [27]. A similar scientific evolution in human medicine led to the inclusion of continued feeding into the standard management protocols for diarrhoea by the WHO in 1988 [12].

The reluctance of veterinarians and farmers to adopt the principle of continued feeding of the diarrhoeic calf led to diverging developments in osmolality of ORS for calves and humans. Commercially available ORS for calves range from isotonic to highly hypertonic, whereas WHO recently changed their recommendation towards a hypotonic formula [13]. Higher osmolality ORS for calves is a reflection of a higher concentration of glucose which is added to provide additional nutritional support. Nonetheless, the provision of high-energy ORS cannot prevent negative energy balance in calves [35] and hypertonic solutions are known to slow abomasal emptying rates compared with isotonic solutions, thereby delaying plasma volume expansion [31].

### Prudent use of antibiotics

There is increasing pressure on the veterinary profession to promote prudent use of antibiotics, noting that indiscriminate use of antibiotics promotes the selection and subsequent proliferation of antibiotic-resistant strains of bacteria [36]. In this context, the benefit of antimicrobial treatment in neonatal calf diarrhoea has been reviewed by Constable [37], who came to the following conclusions:

• Routine use of oral or injectable antibiotics cannot be recommended in calves without systemic illness.

• In calves with diarrhoea and systemic involvement (marked depression, anorexia, fever), the risk of bacteraemia or septicaemia as well as bacterial overgrowth of the small intestine is increased. In such circumstances, administration of broad-spectrum beta-lactam antimicrobials (ceftiofur, amoxicillin or ampicillin), potentiated sulphonamides, or fluoroquinolones (where permitted) is recommended.

• Susceptibility tests of bacteria cultured from faecal samples do not reliably predict treatment outcomes in diarrhoeic calves [37].

### Ancillary treatment

Non-steroidal anti-inflammatory drug therapy with meloxicam has proven effective in improving food intake and weight gain in diarrhoeic calves [38,39]. Flunixin meglumine showed some beneficial effect in experimental calves orally challenged with heat-stable Escherichia coli enterotoxin [40] and in calves with naturally acquired bloody diarrhoea, but not in calves without faecal blood [41].

Additional ancillary treatment has been either suggested or used to treat diarrhoeic calves in the past, but is now either contraindicated (glucocorticoids, motility modifiers) or cannot be supported due to insufficient evidence of efficacy (intestinal protectants, probiotics) [42].

### Diagnostic and specific prevention

For the management of the individual diarrhoeic calf, the knowledge of infectious agents involved is of little value. If specific preventive measures are considered, faecal samples from untreated calves early in the course of clinical disease can be submitted for laboratory analyses [43]. Care has to be taken with the interpretation of results, since the enteropathogens most commonly implicated in calf diarrhoea outbreaks (rotavirus, coronavirus, pathogenic strains of *Escherichia coli*, cryptosporidia, *Salmonella *spp.) can also be found in faecal samples from healthy calves and in calves from farms without diarrhoea problem [8-11]. Postmortem examination of calves dying or euthanased in the acute stage of disease can be beneficial especially for the diagnosis of outbreaks of salmonellosis [44].

Rotavirus and cryptosporidia are the most frequently identified infectious agents in faecal samples from diarrhoeic calves in Ireland [43] and elsewhere [45]. Vaccination is the only prophylactic measure available against rotavirus infection. Vaccination of the dam before calving has been used to enhance the content of rotavirus specific antibodies in colostrum [46]. Commercially available vaccines usually also contain coronavirus and *E. coli *F5 antigen. There is no doubt that modern vaccines are able to increase the level of specific antibodies in serum and milk of vaccinated cows as well as in serum of calves that have ingested colostrum from vaccinated dams [47-49]. However, evidence as to clinical efficacy in naturally acquired diarrhoea is either not available for all vaccines or is conflicting. Using the same vaccine, LeRousic et al. [50] found a reduction in severity of diarrhoea in calves born from vaccinated cows, whereas Kohara et al. [47] failed to find any clinical effect. In either case, available data are not sufficient to assess the economic benefit associated with vaccine usage. Oral vaccination of newborn calves is not effective [51]. In herds endemically infected with *Salmonella *spp., vaccination of the dams prior to calving can be considered [52].

Halofuginone has a demonstrated cryptosporidiostatic effect and is licensed for prevention and treatment of cryptosporidiosis in calves. In a recent review, this substance was found to be beneficial for prophylactic use in cases with severe cryptosporidium-associated diarrhoea. However, data were insufficient to evaluate therapeutic efficacy [53]. In a study on an Irish farm, halofuginone was effective in reducing clinical signs of cryptosporidiosis and environmental contamination [54]. However, it did not delay the onset of diarrhoea or reduce the risk of infection in group-housed calves. The use of halofuginone should be combined with hygienic measures and improvement of the husbandry management system.

## Eimeriosis

Infection with *Eimeria *spp. has a high prevalence in cattle, especially calves and yearlings [55]. Clinical coccidiosis is most often caused by infection with *E. zuernii *or *E. bovis*, generally linked with conditions of high infectious pressure [56,57]. In contrast, infection with other *Eimeria *spp., as well as more pathogenic species but under conditions of low infectious pressure, lead to subclinical coccidiosis [55]. *E. alabamensis *has been reported in outbreaks of watery diarrhoea in calves on pasture in northern Europe [58,59]. The economic impact of clinical, but also subclinical, coccidiosis on the farming industry is considerable, due to both the cost of treatment and impaired performance of affected animals [60]. The general conditions of animal husbandry discussed previously (e.g. housing hygiene, ventilation, immunocompromising factors) each contribute to infection risk and should be critically assessed during coccidiosis outbreaks [61].

Individual animal testing is of limited value because *Eimeria *spp. are frequently found in the faeces of healthy calves [9,55]. To relate clinical observations of diarrhoea to infection with *Eimeria *spp., it is recommended that faecal samples are collected and examined from several animals in a clinically affected group, followed by differentiation of oocysts to the species level [61].

In clinical coccidiosis, the development of diarrhoea is caused by the late stages of the life cycle (second merogony and especially gamogony [62]. Therapeutic intervention at this stage is of limited value and therefore emphasis should be given to metaphylactic treatment in outbreak situations or prophylactic treatment of at-risk groups [63,64].

## Conclusions

Diarrhoea is generally the most common cause of morbidity and mortality in pre-weaned calves. A range of measures are critical to disease prevention, including colostrum management and subsequent nutrition (Lorenz [5]et al., 2011b). Oral rehydration therapy, continued milk feeding and prudent use of antibiotics are each important in the successful management of neonatal calf diarrhoea. Vaccines for neonatal calf diarrhoea are available, however, efficacy reports are variable and data on economic benefit are lacking.

## Conflict of interest statement

None of the authors of this paper has a financial or personal relationship with other people or organisations that could inappropriately influence or bias the content of the paper. The Technical Working Group includes employees of Pfizer Inc. (CC) and Volac Ireland (LG); these companies played no role in the design, development or journal submission of this review series.

## Authors' contributions

IL drafted the manuscript and compiled the literature. All authors made substantial inputs to the review, critically discussed the progressing manuscript and approved the final manuscript.
